# Supplementary material for: Functional Study on the Key Gene LaLBD37 Related to the Lily Bulblets Formation
Source: Int J Mol Sci. 2024 Aug 30;25(17):9456. doi: 10.3390/ijms25179456 (PMC11395201; doi:10.3390/ijms25179456)
Supplement: Supplementary file 1 [file ijms-25-09456-s001.zip › ijms-3155983-supplementary.pdf]

supplementary material

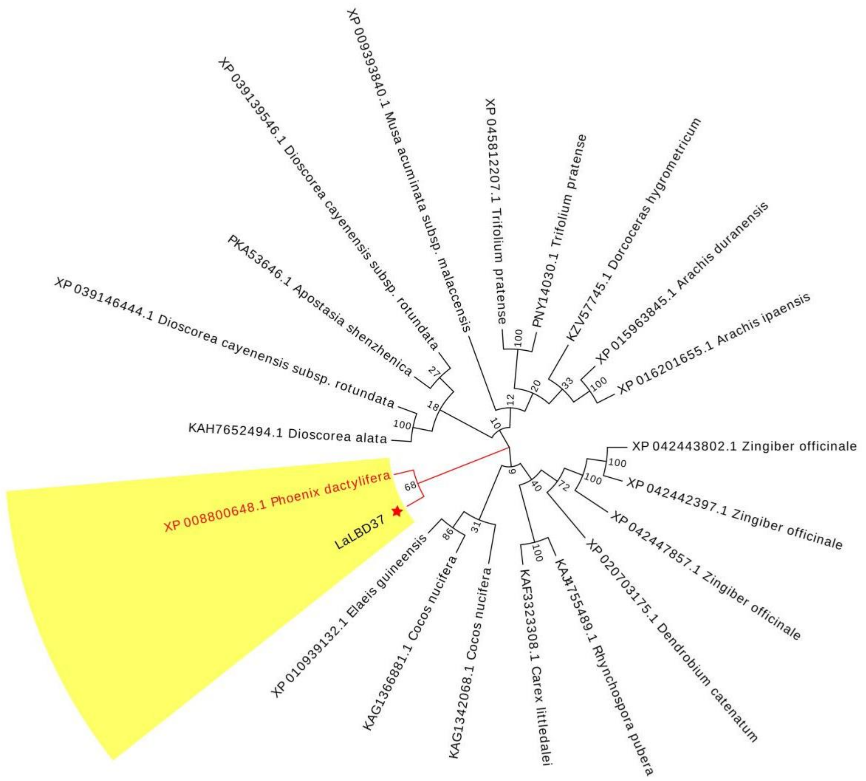

Figure S1. Phylogenetic tree analysis of LaLBD37. Calculate the evolutionary distance using p-distance and perform cluster analysis on the amino acid sequences of LaLBD37 and the LBD family in other species based on the number of amino acid differences at each locus.

Table S1. The name and sequence of primer in this study

Primer name	Sequence (5'-3')
	Gene cloning of *LaLBD37*
LaLBD37-F	ATGAGCTGCAATGGATGCCGAGTGC
LaLBD37-R	GACAAAGAGATTCAACAGTCTAGGTTGCC
	Subcellular localization of *LaLBD37*
LaLBD37-*XhoI*	CATTTACGAACGATACTCGAGATGAGCTGCAATGGATGC
LaLBD37-*SalI*	CACCATCACTAGTACGTCGACGACAAAGAGATTCAACAGTCTAGGTTGCC
	Detection of transcriptional self-activation activity in *LaLBD37*
F-pGBKT7- LaLBD37-A	CATGGAGGCCGAATTCATGAGCTGCAATGGATGC
R-pGBKT7- LaLBD37-A	GCAGGTCGACGGATCCGACAAAGAGATTCAAC
F-pGBKT7- LaLBD37-N	CATGGAGGCCGAATTCATGAGCTGCAATGGATGCCGAGTGC
R-pGBKT7- LaLBD37-N	GCAGGTCGACGGATCCGCCGCCGCGGAGAACTGTCTTAAC
F-pGBKT7- LaLBD37-C	CATGGAGGCCGAATTCACGCCGCGCCCGCTGC
R-pGBKT7- LaLBD37-C	GCAGGTCGACGGATCCGACAAAGAGATTCAACAGTCTAG
	Construction of *LaLBD37* Overexpression Vector

LaLBD37-XbaI	CCAAATCGACTCTAGTCTAGAATGAGCTGCAATGGATGC
*LaLBD37-Sal*I	CACTAGTATTTAAATGTCGACGACAAAGAGATTCAACAGTCTAGGTTGCC
	Real time fluorescence quantitative detection of transformed plants
Q-LaLBD37-F	CTGCGGGCGTACGATCAACC
Q-LaLBD37-R	CTGTCTTAACCGCAGCCTGG
LlTIP41-F	CGAAGCCAGAAACGGAGAAGAAT
LlTIP41-R	GGGTAGGGTGGATTGGGAAGA
